# Associação entre sintomas, veias varicosas e refluxo na veia safena magna ao eco-Doppler

**DOI:** 10.1590/1677-5449.005216

**Published:** 2017

**Authors:** Amélia Cristina Seidel, Mariana Baldini Campos, Raquel Baldini Campos, Dérica Sayuri Harada, Robson Marcelo Rossi, Pedro Cavalari, Fausto Miranda

**Affiliations:** 1 Universidade Estadual de Maringá – UEM, Departamento de Medicina, Maringá, PR, Brasil.; 2 Universidade Estadual de Campinas – UNICAMP, Campinas, SP, Brasil.; 3 Universidade de São Paulo – USP, São Paulo, SP, Brasil.; 4 Universidade Estadual de Maringá – UEM, Departamento de Cirurgia, Maringá, PR, Brasil.; 5 Universidade Federal de São Paulo – UNIFESP, Escola Paulista de Medicina, Departamento de Cirurgia, São Paulo, SP, Brasil.

**Keywords:** refluxo venoso, veia safena, ultrassonografia Doppler em cores, insuficiência venosa, membros inferiores

## Abstract

**Contexto:**

A doença venosa crônica requer avaliação clínica, quantificação dos efeitos hemodinâmicos e definição da distribuição anatômica para decisão diagnóstica e tratamento.

**Métodos:**

Estudo prospectivo realizado em 2015 com amostra de 1.384 pacientes (2.669 membros) com idade entre 17 e 85 anos, sendo 1.227 do sexo feminino. Nas respostas do questionário aplicado, os sintomas pesquisados eram dor, cansaço, sensação de peso, queimação, câimbras e formigamento. Para a formação dos grupos, foi considerado o número de membros, distribuídos em relação ao gênero, ao índice de massa corporal e à idade. Após a definição grupos e a realização do eco-Doppler para estudo da veia safena magna (VSM), os pacientes foram distribuídos em três grupos (I: sintomas presentes e varizes ausentes, II: sintomas ausentes e varizes presentes e III: sintomas presentes e varizes presentes). A análise estatística utilizou o teste qui-quadrado ou exato de Fisher para verificar a homogeneidade entre os grupos. Em caso de associação com significância de 5%, foi calculada a razão de chances.

**Resultados:**

Para ambos os gêneros, foi observada chance de insuficiência da VSM 11,2 vezes maior no grupo III. Por sua vez, os casos de obesidade mórbida ocorreram 9,1 vezes mais no mesmo grupo. Além disso, pacientes na faixa etária entre 30 e 50 anos desse grupo apresentaram chance de insuficiência da VSM 43,1 vezes maior.

**Conclusões:**

A insuficiência da VSM foi significantemente mais frequente no grupo III, tanto globalmente como considerando apenas os casos de obesidade mórbida e a faixa etária mais elevada.

## INTRODUÇÃO

A doença venosa crônica (DVC) é caracterizada pela presença de insuficiência valvar em veias superficiais, perfurantes ou profundas, obstrução do sistema profundo e insuficiência da bomba muscular na panturrilha. Sua presença requer avaliação clínica da gravidade, quantificação de seus efeitos hemodinâmicos e uma melhor definição de sua distribuição anatômica.

Detectar e quantificar o refluxo são importantes medidas para diagnóstico e tratamento^1^. Com o desenvolvimento dos métodos não invasivos como o eco-Doppler, o refluxo tem sido identificado em taxas crescentes nas safenas, muitas vezes em pacientes assintomáticos, embora a um ritmo menor do que em pacientes com doença venosa^2^.

A doença varicosa afeta 1/3 da população, com impacto na qualidade de vida e nos custos de saúde. A veia safena magna (VSM) está envolvida na maior parte dos casos. Suas manifestações são consequência da sobrecarga de volume e da hipertensão nas veias cutâneas causadas por distensão da parede, incompetência valvular, anormalidade do fluxo sanguíneo e fenômenos secundários como alergia e inflamação^3^. No entanto, não há sintomas específicos e outras causas ou doenças podem ser confundidas com insuficiência venosa.

Na maioria dos pacientes, nota-se que a dor é pior após períodos prolongados em pé ou sentado. Desconforto e edema de tornozelo são menos proeminentes no início do dia e mais incômodos no final do dia.

O eco-Doppler é utilizado para determinar a presença de doença funcional que pode estar associada com a presença de dilatações venosas, incluindo telangiectasias, varizes e alterações na pele^1^.

Rotineiramente o refluxo é avaliado no sistema superficial e nas veias perfurantes, o que é seguido da avaliação do refluxo venoso profundo, etapa fundamental para completar o diagnóstico, particularmente em pacientes com edema e danos à pele^4^^,^ 5.

Estudos que associam o refluxo venoso diagnosticado pelo eco-Doppler com a presença ou ausência de sintomas demonstraram que esse exame é útil para identificar a doença venosa em estágios iniciais e, dessa forma, orientar tratamentos adequados.

O objetivo deste trabalho é verificar a presença de associação entre a incidência de refluxo na VSM ao exame de eco-Doppler e a presença de veias varicosas em membros inferiores em pacientes com diagnóstico clínico de insuficiência venosa.

## MÉTODO

O estudo é do tipo transversal investigativo, tendo sido realizado por meio de fichas cadastrais relativas a todos os pacientes voluntários atendidos nos 12 meses de coleta (ano de 2015). O protocolo do estudo foi aprovado pelo Comitê Permanente de Pesquisa em Seres Humanos da UEM (COPEP), processo número 34386814.5.

Foram excluídos os pacientes com história de trombose venosa profunda, doença arterial periférica, operação prévia de varizes, gestantes e portadores de malformação vascular. Também não foram considerados pacientes pertencentes às classes clínicas C_5_ e C_6,_ porque havia um número muito reduzido desses pacientes, visto que a maioria tinha história de trombose profunda ou de operação.

Para este estudo foi utilizada uma amostra sequencial de 1.384 pacientes com idade entre 17 e 85 anos, sendo 1.227 do sexo feminino e 157 do sexo masculino, totalizando 2.669 membros. O cálculo para estimar prevalência, considerando uma população de tamanho desconhecido, com prevalência desconhecida, a um nível de significância de 5% e um erro de no máximo 3%, é de no mínimo 1067 pacientes.

Os dados relativos à anamnese e exame físico foram anotados em protocolo pré-estabelecido. Também foi aplicado um questionário sobre sintomas da DVC cujas respostas eram espontâneas, não sendo apresentadas alternativas, e incluíram mais frequentemente a presença de dor, cansaço, sensação de peso, queimação, câimbras e formigamento. Em relação ao exame físico, foi anotado peso, altura e calculado o índice de massa corporal (IMC) de todos os pacientes. Para distribuição dos grupos (I: sintomas presentes e varizes ausentes, II: sintomas ausentes e varizes presentes e III: sintomas presentes e varizes presentes), foi levada em conta a presença de varizes a partir da classe clínica C_2_.

O eco-Doppler foi realizado de acordo com a literatura, com os pacientes em decúbito dorsal para avaliar o sistema venoso profundo e em posição ortostática para análise do sistema superficial, utilizando-se transdutores lineares de 5 a 7 MHz e convexos de 2 a 3 MHz para os obesos. Para as veias superficiais, o refluxo foi considerado como fluxo retrógrado com tempo de refluxo maior que 500 ms.

Devido ao fato de um mesmo paciente apresentar membros em classes clínicas diferentes, foi considerado, para a formação dos grupos, o número de membros, e estes foram distribuídos inicialmente em relação aos estratos: gênero, IMC (< 25 e ≥ 25 para mulheres; < 30 e ≥ 30 para homens) e faixa etária (< 30, 30-50 e > 50 anos). Somente após essa distribuição foram formados os grupos para pesquisa da insuficiência da VSM de acordo com a presença ou ausência de sintomas de DVC e varizes.

Para análise estatística, foram utilizados o teste qui-quadrado ou, conforme a situação, o teste exato de Fisher para verificar a homogeneidade entre grupos. Em caso de associação ao nível de significância de 5% (p < 0,05), foi calculada a razão de chances (*odds ratio*, OR)^6^.

## RESULTADOS

Considerando-se a amostra global, na Tabela 1 estão listados os grupos com seus respectivos números de membros que apresentavam insuficiência da VSM.

**Tabela 1:** Distribuição dos membros nos diferentes subgrupos amostrais com VSM com refluxo e VSM sem refluxo.

	**Nº membros**	**%**	**VSM insuficiente**			**VSM normal**		
			**Grupo I**	**Grupo II**	**Grupo III**	**Grupo I**	**Grupo II**	**Grupo III**
Amostra total	2.669	100,0	52	95	379	778	765	600
Homens	294	11,0	4	37	67	52	56	78
Mulheres	2.375	89,0	48	58	312	726	709	522
Idade (masculino)								
< 30	29	9,9	0	6	5	11	1	6
30-50	125	42,5	0	12	33	30	27	23
> 50	140	47,6	2	19	31	13	28	47
Idade (feminino)								
< 30	309	13,0	5	4	14	114	139	33
30-50	1.235	52,0	19	27	142	384	387	276
> 50	831	35,0	24	27	156	228	183	213
IMC (masculino)								
< 30	220	75,0	4	31	46	41	41	57
≥ 30	74	25,0	1	6	20	10	15	22
IMC (feminino)								
< 25	1.280	54,0	23	29	120	424	435	249
≥ 25	1.095	46,0	25	29	192	302	274	273

VSM: veia safena magna; IMC: índice de massa corporal.

Pela avaliação estatística da amostra total, observou-se uma diferença significante (p < 0,01) entre os grupos em relação à presença de insuficiência da VSM, a qual esteve presente em 52 (1,9%) dos membros no grupo I, 95 (3,6%) no grupo II e 379 (14,2%) no grupo III. Verificou-se que a probabilidade de insuficiência da VSM no grupo II foi aproximadamente duas vezes maior do que no grupo I (grupo de referência) e analogamente, 9,5 vezes maior no grupo III. Por outro lado, comparando-se os grupos II e III, conclui-se que o grupo III teve 5,1 vezes mais chance de apresentar insuficiência da VSM se comparado ao grupo II (referência) (Tabela 2).

**Tabela 2:** Distribuição dos membros nos diferentes grupos da amostra de acordo com a presença ou não de sintomas, varizes e insuficiência da VSM.

**Amostra**	**Nº membros**		**Grupos**		***p-valor***
		**I**	**II**	**III**	
Geral	2.669	52 (6,3%)	95 (11,1%)	379 (38,7%)	< 0,01^*^
OR		ref	1,9	9,5	
			ref	5,1	

* Teste exato de Fisher. ref: referência; VSM: veia safena magna; OR: odds ratio.

Na amostra total dos pacientes do sexo masculino, a insuficiência da VSM esteve presente em 4 (7,1%) dos membros no grupo I, 37 (39,8%) no grupo II e 67 (42,5%) no grupo III. Essa insuficiência, tendo o grupo I como referência, foi 8,6 vezes maior no grupo II e 11,2 vezes maior no grupo III. No subgrupo dessa amostra com IMC < 30, a chance de insuficiência da VSM no grupo II foi 7,8 vezes maior e 8,3 vezes maior no grupo III do que no grupo I (grupo de referência), respectivamente. Na presença de IMC ≥ 30, não houve significância estatística na comparação entre os grupos I e II, mas houve uma chance 9,1 vezes maior no grupo III se comparado ao grupo I (grupo de referência). Ainda com base nos valores apresentados na Tabela 3, a chance de insuficiência da VSM naqueles com menos de 30 anos foi 66 vezes maior no grupo II que no grupo I (grupo de referência) e não apresentou significância estatística na comparação entre os grupos I e III. Nos pacientes com idade entre 30 e 50 anos, a chance foi 13,3 vezes maior no grupo II e 43,1 vezes maior no grupo III se comparados ao grupo I.

**Tabela 3:** Presença de sintomas e/ou varizes e suas relações com a insuficiência da VSM em homens.

**Amostras**	**Nº membros**	**Grupos**			***p-valor***
		**I**	**II**	**III**	
Geral	294	4 (7,1%)	37 (39,8%)	67 (42,5%)	< 0,01
OR		ref	8,6	11,2	
IMC < 30	220	4	31	46	< 0,01
OR		ref	7,8	8,3	
IMC ≥ 30	74	1	6	20	0,042^*^
OR		ref	ns	9,1	
< 30 anos	29	0	6	5	0,001^*^
OR		ref	66,0	ns	
30-50 anos	125	0	12	33	< 0,01
OR		ref	13,3	43,1	
			ref	3,2	
> 50 anos	140	2	19	31	0,130

* Teste exato de Fisher. ref: referência; ns: não significante; VSM: veia safena magna; OR: odds ratio.

Considerando o conjunto geral de membros do sexo feminino, a insuficiência da VSM esteve presente em 48 (6,2%) dos membros no grupo I, 58 (7,6%) no grupo II e 312 (37,4%) no grupo III. Pode-se observar que não houve diferença estatística na comparação dos grupos I e II considerando-se todos os seus subgrupos. Porém, essa diferença esteve presente na comparação entre os grupos I e III e entre os grupos II e III (Tabela 4).

**Tabela 4:** Presença de sintomas e/ou varizes e suas relações com a insuficiência da VSM em mulheres.

**Amostras**	**Nº membros**	**Grupos**			***p-valor***
		**I**	**II**	**III**	
Geral	2.375	48 (6,2%)	58 (7,6)	312 (37,4%)	< 0,01
OR		ref	ns	9,0	
			ref	7,3	
IMC < 25	1.280	23	29	120	< 0,01
OR		ref	ns	8,9	
			ref	7,2	
IMC ≥ 25	1.095	25	29	192	< 0,01
OR		ref	ns	8,5	
			ref	6,6	
< 30 anos	309	5	4	14	< 0,01^*^
OR		ref	ns	9,7	
			ref	14,7	
30-50 anos	1.235	19	27	142	< 0,01
OR		ref	ns	10,4	
			ref	7,4	
> 50 anos	831	24	27	156	< 0,01
OR		ref	ns	7,0	
			ref	5,0	

* Teste exato de Fisher. ref: referência; ns: não significante; VSM: veia safena magna; OR: *odds ratio*.

## DISCUSSÃO

Foram alocados pacientes a partir da classe clínica C_2_ porque, se fossem incluídas telangiectasias e aranhas vasculares, elas ocorreriam em mais de 50% dos pacientes acima de 40 anos, e muitos destes não têm sintomas e não têm interesse em realizar procedimentos por razões estéticas. Sintomas como peso, dor, edema e prurido podem ser citados, mas nenhum deles é específico de DVC, podendo estar associados a múltiplas etiologias. Para relacioná-los à presença de varizes, é importante levar em conta a localização específica desses sintomas, suas características e fatores precipitantes^2^.

Na anamnese, apesar de não terem sido apresentadas alternativas, houve o cuidado de incluir apenas os sintomas supostamente relacionados à DVC, seguindo as informações da literatura^2^^,^ 7 que demonstram a importância de nominar os sintomas, particularmente em pacientes idosos, nos quais outras condições podem causar dor e edema nas extremidades, como artrite, neuropatia, claudicação, estenose espinhal, insuficiência cardíaca congestiva, insuficiência renal e outras.

Sintomas de varizes não acompanhados de veias varicosas têm sido um dos aspectos mais controversos da angiologia. Sugeriu-se que a fisiopatologia da presença de sintomas de doença venosa sem varizes se deve à redução do tônus da parede venosa e propôs-se o termo flebopatia hipotônica para denominar a condição^8^. Isso pode ter acontecido no grupo I, pois somente 1,9% dos membros tinham insuficiência da VSM.

Em 2013, foi realizado um estudo que investigou a associação do receio de ter varizes e incompetência da VSM não conhecida com a prevalência e os achados de sintomas de varizes em indivíduos saudáveis e pacientes com veias varicosas. Os autores concluíram que indivíduos saudáveis com receio de ter varizes apresentam sintomas com igual frequência que aqueles com incompetência da VSM não conhecida e pacientes com veias varicosas^9^.

Entre vários fatores de risco (obesidade^10^^,^ 11, história familiar^9^, gravidez, trabalhar em pé ou sentado por longos períodos^12^), parece claro que a idade e o sexo feminino se destacam, sendo citados no Projeto Acireale^8^ e por outros autores^13^^,^ 14; entretanto, o sexo feminino não tem sido universalmente considerado um fator de risco positivo^2^. Em um dos poucos estudos que demonstram a associação de veias varicosas e DVC na população geral, o *Edinburgh Vein Study*, observou que a incidência dessas condições não diferiu significativamente entre os sexos, mas afirma que a incidência das veias varicosas aumenta com a idade, história familiar e IMC^15^.

A definição ideal de obesidade é baseada na gordura corporal. Apesar das diferenças encontradas no IMC entre indivíduos de diferentes idades e sexos, a Organização Mundial de Saúde ainda recomenda o uso do IMC para se determinar o índice de obesidade^16^^,^ 17. Há evidências científicas sugerindo que homens e mulheres devam ter diferentes limiares para definir excesso de peso, porque é normal que as mulheres tenham mais gordura que os homens e que os homens tenham mais massa muscular que as mulheres, e músculo pesa mais que gordura^18^. Há estudos sugerindo diferentes “gaps” de IMC adequados entre homens e mulheres, desde 1,4 kg/m^2^ 19, 2 kg/m^2^ 20 ou 5 kg/m^2^ 21. Não há uma única resposta correta.

Devido à discordância encontrada na literatura em relação aos fatores de risco para insuficiência da VSM, optou-se por distribuir os pacientes em diferentes grupos. Primeiro foi considerado o gênero (masculino e feminino) e então, dentro de cada gênero, os pacientes foram redistribuídos em três subgrupos segundo a idade (< 30, 30-50 e > 50 anos) e outros dois quanto ao IMC (com valor de corte para obesidade estipulado em 25 para mulheres e 30 para homens)^22^.

O eco-Doppler foi a escolha para o estudo do sistema venoso, pois o refluxo na VSM tem sido identificado mesmo em pacientes assintomáticos^23^, sendo considerado como o melhor método para avaliar o refluxo em segmentos de veias individuais. O exame foi realizado com o paciente em pé e teve valor de corte para refluxo nas veias superficiais maior do que 500 ms^24^.

Ao investigar a associação entre achados ultrassonográficos e classificação Clinical, Etiology, Anatomy and Physiopathology (CEAP) em um grupo de 1.029 pacientes, um estudo observou que a presença de obstrução no sistema venoso profundo em pacientes com DVC pertencentes à classe clínica C_0_-C_1_ poderia justificar a recomendação para a realização do eco-Doppler em todos os pacientes com sintomas de DVC mas sem sinais clínicos^25^.

No presente estudo, é possível verificar que a insuficiência da VSM está presente em maior porcentagem no grupo III, o qual apresenta sintomas e varizes, corroborando com os dados de outro estudo^1^ que avaliou a associação entre intensidade de refluxo na junção safenofemoral (JSF) e alterações de diâmetro da VSM incompetente. Os autores desse estudo observaram uma correlação entre diâmetro, velocidade e fluxo na VSM e gravidade clínica, de acordo com a CEAP.

Segundo a literatura^26^, um refluxo maior é acompanhado por uma clínica mais pronunciada. Notou-se um aumento da frequência de sintomas e alterações da pele na presença de incompetência da VSM e/ou da JSF, sobretudo quando o refluxo desta se estende até o maléolo. Confirmamos essa observação ao mostrar que pacientes com sintomas de DVC e varizes apresentam maior incidência de refluxo. Além disso, nos casos de refluxo isolado da VSM sem sinais e com sintomas, como ocorreu no grupo I, há baixa porcentagem de insuficiência da VSM.

Contudo, em discordância com esses achados, Chastanet e Pittalugo^26^ mostraram que pacientes com incompetência da VSM sem varizes apresentaram uma alta frequência de sintomas e alterações na pele, o que poderia significar uma forma particular de DVC com deficiência precoce da VSM resultando em aumento da morbidade. O desenvolvimento de incapacidade na JSF parece ser um “ponto-chave”, pois a taxa de alterações tróficas aumenta de 1,7% para 10,6%, de acordo com a funcionalidade da JSF.

Somando os resultados de três trabalhos de epidemiologia realizados nos Estados Unidos, concluiu-se que aproximadamente 15-25% da população tem varizes, com prevalência maior em mulheres e em idosos. Um desses trabalhos^27^, realizado na área de San Diego em 2003, que avaliou 2.211 participantes com eco-Doppler para determinar a presença de doença funcional e correlacioná-la com a presença de alterações venosas visíveis, detectou uma incidência de veias varicosas em 23,3% da amostra. Além disso, o mesmo estudo também observou que, na avaliação com o eco-Doppler, 19% da amostra total tinha doença funcional superficial. No *National Venous Screening Program* 28, 23% dos 2.234 indivíduos analisados apresentavam varizes. Já no *Tecumseh Community Health Study* 29, o diagnóstico de varizes esteva presente em 25,9% dos participantes do sexo feminino e em 12,9% dos do sexo masculino.

Apesar de não ser um estudo epidemiológico, os resultados encontrados não foram semelhantes aos dos estudos anteriores, isto é, nos grupos em que os pacientes apresentavam varizes (grupo II - assintomáticos, e grupo III - sintomáticos) a incidência de VSM com refluxo foi de 11,1% e 38,7% respectivamente.

Os resultados deste trabalho estão em concordância com os autores^26^ que descreveram ser a presença de veias varicosas sem refluxo da VSM mais frequente em jovens e observaram que a incompetência da JSF e VSM com sintomas ocorre mais em idosos.

De acordo com esses autores e com base nos achados deste estudo, acreditamos que deve ser realizado o tratamento precoce da insuficiência venosa antes que ocorram sintomas e deterioração fisiológica.

## CONCLUSÃO

Nos pacientes que apresentaram varizes nos membros inferiores visíveis e sintomáticas (grupo III), houve globalmente maior ocorrência de insuficiência da VSM (p < 0,01), considerando ambos os gêneros. O mesmo ocorreu na presença de IMC ≥ 30 (masculino) e ≥ 25 (feminino). A insuficiência da VSM também apresentou incidência significantemente maior na faixa etária entre 30 e 50 anos. Na faixa etária > 50 só ocorreu incidência maior no gênero feminino, enquanto que no masculino não houve significância. Porém, devemos levar em conta que o número de pacientes do sexo masculino era pequeno nessa faixa etária.
