# Supplementary material for: Associação entre sintomas, veias varicosas e refluxo na veia safena magna ao eco-Doppler
Source: J Vasc Bras. 2017 Jan-Mar;16(1):4–10. [Article in Portuguese] doi: 10.1590/1677-5449.005216 (PMC5829685; doi:10.1590/1677-5449.005216)
Supplement: Translation

# Translation: Associations between symptoms and varicose veins and great saphenous vein reflux seen on Doppler ultrasonography

**Authors:** Amélia Cristina Seidel, Mariana Baldini Campos, Raquel Baldini Campos, Dérica Sayuri Harada, Robson Marcelo Rossi, Pedro Cavalari, Fausto Miranda

**Affiliations:** 1 Universidade Estadual de Maringá – UEM, Departamento de Medicina, Maringá, PR, Brazil.; 2 Universidade Estadual de Campinas – UNICAMP, Campinas, SP, Brazil.; 3 Universidade de São Paulo – USP, São Paulo, SP, Brazil.; 4 Universidade Estadual de Maringá – UEM, Departamento de Cirurgia, Maringá, PR, Brazil.; 5 Universidade Federal de São Paulo – UNIFESP, Escola Paulista de Medicina, Departamento de Cirurgia, São Paulo, SP, Brazil.

**Keywords:** venous reflux, saphenous vein, color Doppler ultrasonography, venous insufficiency, lower limbs

## Abstract

**Background:**

Chronic venous disease demands clinical assessment, quantification of hemodynamic effects, and definition of anatomic distribution before diagnostic and treatment decisions can be made.

**Methods:**

This is a prospective study conducted in 2015 with a sample of 1,384 patients (2,669 limbs) aged from 17 to 85 years, 1,227 of whom were female. The most common symptoms reported in response to the questionnaire were pain, tiredness, feelings of heaviness, burning, cramps, and tingling. Subsets were formed on the basis of number of limbs distributed by sex, body mass index, and age. After definition of subsets, Doppler ultrasonography was used to conduct examinations of the great saphenous vein (GSV) and patients were distributed into three clinical groups (I: symptoms present and varicose veins absent, II: symptoms absent and varicose veins present and III: symptoms present and varicose veins present). Statistical analysis employed the chi-square test or Fisher’s exact test to test for homogeneity between groups. When associations significant to 5% were detected, odds ratios were calculated.

**Results:**

For both sexes, the chance of GSV insufficiency was 11.2 times greater in group III. Among cases with morbid obesity, the chance was 9.1 times greater in the same group. Additionally, patients in this group with ages ranging from 30 to 50 years exhibited a 43.1 times greater chance of GSV insufficiency.

**Conclusions:**

Insufficiency of the GSV was significantly more frequent in group III, both overall and when considering only cases with morbid obesity, or cases in older age groups.

## INTRODUCTION

Chronic venous disease (CVD) is characterized by valve incompetence in superficial, perforating, or deep veins, obstruction of the deep system, and insufficiency of the calf muscle pump. When present, it is necessary to conduct clinical assessment of severity, quantification of hemodynamic effects, and arrive at a better definition of its anatomic distribution.

Detection and quantification of reflux are important steps towards diagnosis and treatment.^1^ Development of noninvasive methods such as Doppler ultrasonography has led to increasing rates of identification of reflux in the saphenous veins, very often in patients who are asymptomatic, although less frequently than among patients with venous disease.^2^

Varicose disease affects 1/3 of the population, impacting on quality of life and increasing healthcare costs. The great saphenous vein (GSV) is involved in the majority of cases. Its manifestations are a consequence of volume overload and hypertension in cutaneous veins caused by distension of the walls, valve incompetence, abnormal blood flow, and secondary phenomena such as allergy and inflammation.^3^ However, there are no specific symptoms and other causes or diseases can be confused with venous insufficiency.

In the majority of patients, it is observed that pain is worse after prolonged periods standing up or sitting down. Discomfort and edema of the ankle are less common at the start of the day and cause greater inconvenience at the end of the day.

Doppler ultrasonography is used to detect the presence of functional disease that may be associated with presence of venous dilation, including telangiectasias, varicose veins and skin abnormalities.^1^

Reflux is routinely assessed in the superficial system and perforating veins, which is then followed up with assessment of deep venous reflux, which is an indispensable step in completing a diagnosis, particularly in patients who have edema and skin involvement.^4^^,^ 5

Studies that associate venous reflux diagnosed with Doppler ultrasonography with presence or absence of symptoms have demonstrated that this method of examination is useful for identifying venous disease while in the initial stages and, therefore, to provide guidance for choosing the most appropriate treatments.

The objective of this study is to test for an association between incidence of GSV reflux detected by Doppler ultrasonography examination and presence of varicose veins in the lower limbs of patients with clinical diagnoses of venous insufficiency.

## METHOD

This is a cross-sectional, investigative study, conducted by analysis of records for all patients who were seen during a 12-month data-collection period (2015) and volunteered to take part. The protocol for the study was approved by the UEM’s Permanent Human Research Commission, under process number 34386814.5.

Patients with a history of deep venous thrombosis, peripheral arterial disease, previous operations on varicose veins, expectant mothers, and patients with vascular malformations were all excluded. Patients were also excluded if they had been diagnosed in clinical classes C_5_ or C_6_, because there were very few such patients, since the majority had a history of deep thrombosis or venous operations.

The data used for this study is from a sequential sample of 1,384 patients, aged from 17 to 85 years, 1,227 of whom were female and 157 of whom were male, and relates to a total of 2,669 limbs. A calculation conducted to estimate prevalence, considering a population of unknown size, with unknown prevalence, to a significance level of 5%, and a maximum error of 3%, indicated that the sample should contain a minimum of 1,067 patients.

Data from patient histories and physical examinations were recorded on a pre-prepared chart. An open questionnaire on the symptoms of CVD was also administered, eliciting spontaneous responses with no multiple choice options. The most frequently reported symptoms were pain, tiredness, feelings of heaviness, burning, cramps, and tingling. From the physical examination findings, weight and height were recorded and used to calculate body mass index (BMI) for all patients. Patients were allocated to clinical groups (I: symptoms present and varicose veins absent, II: symptoms absent and varicose veins present, or III: symptoms present and varicose veins present) taking into consideration varicose veins graded as clinical class C_2_ or higher.

Doppler ultrasonography was conducted as recommended in the literature, with patients in decubitus dorsal to examine the deep vein system and standing upright for analysis of the superficial system, using 5 to 7 MHz linear transducers or 2 to 3 MHz convex transducers for obese patients. Reflux in superficial veins was defined as retrograde flow if reflux time was greater than 500 ms.

The number of limbs was considered for formation of subsets because the same patient could have different limbs graded in different clinical classes and were initially distributed according to the following strata: sex, BMI (< 25 and ≥ 25 for women; < 30 and ≥ 30 for men), and age (< 30, 30-50, or > 50 years). Once these subsets had been formed, limbs were allocated to clinical groups for analysis of GSV incompetence according to presence or absence of symptoms of CVD and of varicose veins.

Statistical analysis was conducted using the chi-square test or, depending on the situation, Fisher’s exact test, to test for homogeneity between groups. When associations significant to 5% (p < 0.05) were detected, odds ratios (OR) were calculated.^6^

## RESULTS

Considering the sample as a whole, Table 1 lists the clinical groups by presence or absence of GSV incompetence, showing the number of limbs in each.

**Table 1:** Distribution of limbs in different subsets of the sample, with GSV reflux and without GSV reflux.

	**No. limbs**	**%**	**GSV incompetent**			**GSV normal**		
			**Group I**	**Group II**	**Group III**	**Group I**	**Group II**	**Group III**
Entire sample	2,669	100.0	52	95	379	778	765	600
Men	294	11.0	4	37	67	52	56	78
Women	2,375	89.0	48	58	312	726	709	522
Age (males)								
< 30	29	9.9	0	6	5	11	1	6
30-50	125	42.5	0	12	33	30	27	23
> 50	140	47.6	2	19	31	13	28	47
Age (females)								
< 30	309	13.0	5	4	14	114	139	33
30-50	1,235	52.0	19	27	142	384	387	276
> 50	831	35.0	24	27	156	228	183	213
BMI (males)								
< 30	220	75.0	4	31	46	41	41	57
≥ 30	74	25.0	1	6	20	10	15	22
BMI (females)								
< 25	1,280	54.0	23	29	120	424	435	249
≥ 25	1,095	46.0	25	29	192	302	274	273

GSV: great saphenous vein; BMI: body mass index.

The statistical analysis for the whole sample reveals a significant difference (p < 0.01) between the three groups in terms of GSV incompetence, which was observed in 52 (1.9%) limbs in group I, 95 (3.6%) limbs in group II, and 379 (14.2%) limbs in group III. It was found that the chance of GSV incompetence in group II was approximately twice that of group I (the reference group) and, analogously, 9.5 times greater in group III. On the other hand, comparison of group II with group III indicated that group III had a 5.1 times greater chance of GSV incompetence when compared with group II (the reference group) (Table 2).

**Table 2:** Distribution of limbs in different subsets of the sample by presence or absence of symptoms, varicose veins, and GSV incompetence.

**Sample**	**No. limbs**		**Groups**		***p*-value**
		**I**	**II**	**III**	
Total	2,669	52 (6.3%)	95 (11.1%)	379 (38.7%)	< 0.01^*^
OR		ref	1.9	9.5	
			ref	5.1	

* Fisher’s exact test. ref: reference group; GSV: great saphenous vein; OR: odds ratio.

In the male subset of the sample, GSV incompetence was present in 4 (7.1%) limbs in group I, 37 (39.8%) limbs in group II, and 67 (42.5%) limbs in group III. The likelihood of incompetence, taking group I as the reference, was 8.6 times greater in group II and 11.2 times greater in group III. In the subset of males with BMI < 30, the chance of GSV incompetence was 7.8 times greater in group II and 8.3 times greater in group III in comparison to group I (reference group), respectively. In the male subset with BMI ≥ 30, the comparison between groups I and II did not detect statistical significance, but there was a 9.1 times greater likelihood in group III when compared with group I (reference group). Still with relation to the values for male patients shown in Table 3, the likelihood of GSV incompetence among patients less than 30 years old was 66 times greater in group II than in group I (reference group), while there was no statistically significant difference in the comparison between groups I and III. Among male patients aged 30 to 50, likelihood was 13.3 times greater in group II and 43.1 times greater in group III, in comparison to group I.

**Table 3:** Presence of symptoms and/or varicose veins and their relationships with GSV incompetence in men.

**Samples**	**No. limbs**	**Groups**			***p*-value**
		**I**	**II**	**III**	
Overall	294	4 (7.1%)	37 (39.8%)	67 (42.5%)	< 0.01
OR		ref	8.6	11.2	
BMI < 30	220	4	31	46	< 0.01
OR		ref	7.8	8.3	
BMI ≥ 30	74	1	6	20	0.042^*^
OR		ref	ns	9.1	
< 30 years	29	0	6	5	0.001^*^
OR		ref	66.0	ns	
30-50 years	125	0	12	33	< 0.01
OR		ref	13.3	43.1	
			ref	3.2	
> 50 years	140	2	19	31	0.130

* Fisher’s exact test. ref: reference group; ns: not significant; GSV: great saphenous vein; OR: odds ratio.

Considering the subset of all female limbs, GSV incompetence was present in 48 (6.2%) of the limbs in group I, 58 (7.6%) limbs in group II, and 312 (37.4%) limbs in group III. It can be observed that there were no statistical differences in the comparison between groups I and II in any of their subsets. However, differences were detected between groups I and III and between groups II and III (Table 4).

**Table 4:** Presence of symptoms and/or varicose veins and their relationships with GSV incompetence in women.

**Samples**	**No. limbs**	**Groups**			***p*-value**
		**I**	**II**	**III**	
Overall	2,375	48 (6.2%)	58 (7.6)	312 (37.4%)	< 0.01
OR		ref	ns	9.0	
			ref	7.3	
BMI < 25	1,280	23	29	120	< 0.01
OR		ref	ns	8.9	
			ref	7.2	
BMI ≥ 25	1,095	25	29	192	< 0.01
OR		ref	ns	8.5	
			ref	6.6	
< 30 years	309	5	4	14	< 0.01^*^
OR		ref	ns	9.7	
			ref	14.7	
30-50 years	1,235	19	27	142	< 0.01
OR		ref	ns	10.4	
			ref	7.4	
> 50 years	831	24	27	156	< 0.01
OR		ref	ns	7.0	
			ref	5.0	

* Fisher’s exact test. ref: reference group; ns: not significant; GSV: great saphenous vein; OR: odds ratio.

## DISCUSSION

Patients were only recruited from clinical classes C_2_ and above, because more than 50% of patients aged 40 or over have telangiectasias and/or thread veins and many of these people do not have symptoms and are not interested in undergoing procedures for esthetic reasons. Symptoms such as heaviness, pain, edema, and itching may be reported, but none of these are specific to CVD and can be associated with several different etiologies. To confirm that they are related to the presence of varicose veins, it is important to consider the specific sites of these symptoms, their characteristics and the factors that precipitate them.^2^

During the anamnesis, although response options were not provided, care was taken to only include symptoms supposedly related to CVD, according to information available in the literature^2^^,^ 7 which demonstrates the importance of choosing symptoms, particularly in elderly patients, in whom other conditions such as arthritis, neuropathy, claudication, spinal stenosis, congestive heart failure, renal failure, and others, can cause pain and edema in the extremities.

Symptoms of varicose veins in the absence of varicose veins have become one of the most controversial issues in angiology. It has been suggested that the pathophysiology of the presence of symptoms of venous disease in the absence of varicose veins is caused by a reduction in the tone of the vein wall and the term hypotonic phlebopathy has been proposed to refer to the condition.^8^ This may have occurred in group I, since just 1.9% of the limbs had GSV incompetence.

In 2013, a study was conducted that investigated associations between fears of getting varicose veins and unknown GSV incompetence and prevalence and findings of symptoms of varicose veins in healthy individuals and patients with varicose veins. The authors concluded that healthy people with fears of getting varicose veins exhibit symptoms with the same frequency as people with unknown GSV incompetence and patients with varicose veins.^9^

Among many other risk factors (obesity,^10^^,^ 11 family history,^9^ pregnancy, working standing or sitting for long periods^12^), it seems clear that age and female sex stand out, and have been identified by the Acireale project^8^ and other authors.^13^^,^ 14 However, female sex has not universally been considered a positive risk factor.^2^ One of the few studies that has analyzed the association between varicose veins and CVD in the general population, the Edinburgh Vein Study, observed that the incidence of these conditions does not differ significantly between the sexes, but stated that incidence of varicose veins does increase with age, family history, and BMI.^15^

The ideal definition of obesity is based on body fat. Despite the differences in BMI observed in individuals of different ages and sexes, the World Health Organization still recommends using BMI to determine obesity rates.^16^^,^ 17 There is scientific evidence to suggest that different cutoffs should be used to define excess weight in men and women, because it is normal for women to have more fat than men and for men to have more muscle mass than women and muscle weighs more than fat.^18^ There are studies that suggest that different “gaps” in BMI between men and women are appropriate, ranging from 1.4 kg/m^2^ 19 through 2 kg/m^2^ 20 to 5 kg/m^2^.^21^ There is no single correct answer.

In view of the disagreement in the literature with relation to the risk factors for GSV incompetence, the decision was taken to distribute the patients into several subsets. The first characteristic considered was sex (male or female) and then, within each sex subset, patients were allocated to three further subsets by age (< 30, 30-50, and > 50 years) and two by BMI (with the cutoff for obesity set at 25 for women and 30 for men).^22^

Doppler ultrasonography was chosen to study the venous system because GSV reflux has been identified in asymptomatic patients,^23^ and this is considered the best method for assessment of reflux in individual vein segments. Examinations were conducted with the patients standing up and the cutoff for reflux in superficial veins was set at > 500 ms.^24^

One study investigated associations between ultrasonographic findings and Clinical Etiology Anatomy Physiopathology (CEAP) classification in a group of 1,029 patients and observed that presence of obstruction in the deep vein systems of patients with CVD graded as clinical classes C_0_-C_1_ could justify a recommendation for Doppler ultrasonography in all patients with symptoms of CVD but without clinical signs.^25^

In the present study, it was observed that GSV incompetence was present in a greater percentage of patients in group III, who had both symptoms and varicose veins, corroborating data from another study^1^ which evaluated the association between intensity of reflux at the saphenofemoral junction (JSF) and changes in the diameter of incompetent GSVs. The authors of that study observed a correlation between diameter, velocity, and flow in the GSV and clinical severity according to the CEAP system.

According to the literature,^26^ greater reflux is accompanied by more pronounced clinical status. An increase has been observed in the frequency of symptoms and skin problems in the presence of GSV and/or JSF incompetence, particularly when reflux extends as far as the malleolus. We have confirmed that observation, showing that patients with symptoms of CVD and varicose veins have a greater incidence of reflux. Additionally, in cases of isolated GSV reflux, in the absence of signs but with symptoms, as in group I, there is a low percentage of GSV incompetence.

However, contradicting these findings, Chastanet and Pittalugo^26^ showed that patients with GSV incompetence but without varicose veins exhibited a high frequency of symptoms and skin abnormalities, which could indicate a specific form of CVD with early GSV deficiency resulting in increased morbidity. Development of JSF incapacity appears to be a key point, since the rate of trophic lesions increases from 1.7% to 10.6%, depending on JSF function.

Taking the results of three epidemiological studies conducted in the United States together, it was concluded that approximately 15-25% of the population has varicose veins, with greater prevalence among women and the elderly. One of these studies,^27^ conducted in the San Diego area in 2003, assessed 2,211 participants with Doppler ultrasonography to determine whether they had functional disease and to correlate it with presence of visible venous changes, detecting an incidence of varicose veins equating to 23.3% of the sample. Additionally, the same study also observed that, when assessed with Doppler ultrasonography, 19% of the total sample had superficial functional disease. In the National Venous Screening Program,^28^ 23% of 2,234 people analyzed had varicose veins. In the Tecumseh Community Health Study,^29^ 25.9% of female participants and 12.9% of males were diagnosed with varicose veins.

While this is not an epidemiological study, the results observed were not similar to those of previous studies to the extent that in the groups in which patients had varicose veins (group II - asymptomatic, and group III - symptomatic) the incidence of GSV with reflux was 11.1% and 38.7% respectively.

The results of this study are in agreement with authors^26^ who have described presence of varicose veins without GSV reflux as being more frequent in younger people and have observed that JSF and GSV incompetence with symptoms are more common among older people.

In agreement with those authors, and on the basis of the findings of this study, we believe that venous insufficiency should be treated early, before symptoms and physiological deterioration occur.

## CONCLUSIONS

Among patients who had visible varicose veins in lower limbs and were symptomatic (group III), there was a greater frequency of GSV incompetence (p < 0.01) in all subsets and both sexes. The frequency of GSV incompetence was also higher in patients with BMI ≥ 30 (male) and ≥ 25 (female) and had a significantly greater incidence in the age group from 30 to 50 years. In the age group > 50, incidence was only higher among females, whereas among males the difference was not significant. However, it should be borne in mind that the number of male patients in this age group was small.
